# Association Between Online Information-Seeking and Adherence to Guidelines for Breast and Prostate Cancer Screening

**DOI:** 10.5888/pcd15.170147

**Published:** 2018-04-19

**Authors:** Hankyul Kim, Christopher Filson, Peter Joski, Silke von Esenwein, Joseph Lipscomb

**Affiliations:** 1 Rollins School of Public Health, Emory University, Atlanta, Georgia; 2 Emory University School of Medicine, Atlanta, Georgia; 3 Winship Cancer Institute of Emory University, Atlanta, Georgia

## Abstract

**Introduction:**

From 2012 through 2014, the US Preventive Services Task Force (USPSTF) recommended biennial mammography for women aged 50 to 75 and recommended against the prostate specific antigen (PSA) test for men of any age, emphasizing informed decision making for patients. Because of time constraints and other patient health priorities, health care providers often do not discuss benefits and risks associated with cancer screening. We analyzed the association between seeking information online about breast and prostate cancer and undergoing mammography and PSA screening.

**Methods:**

We assessed guideline concordance in mammogram and PSA screening, according to USPSTF guidelines for those at average risk for disease. We used data on 4,537 survey respondents from the National Cancer Institute’s Health Information National Trends Survey (HINTS) for 2012 through 2014 to assess online information-seeking, defined as whether people searched for cancer-related information online in the past 12 months. We used HINTS data to construct multivariable logistic regression models to isolate the effect of exposure to online information on the incidence of cancer screening.

**Results:**

After controlling for available covariates, we found no significant association between online information-seeking and guideline-concordant screening for breast or prostate cancer. Significant covariate values suggest that factors related to access to care were significantly associated with conformance to mammography guidelines for women recommended for screening and that physician discussion was significantly associated with nonconformance to guidelines for prostate-specific antigen screening (ie, having a PSA test in spite of the recommendation not to have it). Decomposition of differences between those who sought online information and those who did not indicated that uncontrolled confounders probably had little effect on findings.

**Conclusion:**

We found little evidence that online information-seeking significantly affected screening for breast or prostate cancer in accordance with USPSTF guidelines among people at average risk.

## Introduction

Most cancer screening guidelines incorporate informed decision making as a required element (1–3). To qualify as informed decision making, people must be aware of their cancer risk and discuss the benefits and possible harms of screening with their health care provider (4). Despite the emphasis on the value of informed decision making in guidelines issued by the US Preventive Services Task Force (USPSTF) and other organizations, studies show that few people with average risk for cancer are aware of the ongoing debate about the potential harms associated with some types of cancer screening and may overestimate benefits and underestimate potential risks (5–7). Some studies show that interventions at clinics, with decision aids such as questionnaires and counseling, can increase patient understanding of potential harms of screening and may facilitate discussion between patients and their health care providers (7–11). However, little is known about how people at average risk acquire screening-related information and if and how they use this information in discussions with their providers to arrive at a screening decision.

To characterize the patient-centered issues surrounding informed decision making, we examined the relationship between online information-seeking and adherence to USPSTF recommendations for breast cancer and prostate cancer screening. USPSTF recommends that women aged 50 to 75 and at average risk for breast cancer undergo mammography screening every 2 years (3) and recommends against screening average-risk men with the prostate-specific antigen (PSA) test (during the 2012–2014 timeframe relevant to this study) (1). We hypothesized that people who engaged in online information-seeking would be more likely to adhere to USPSTF screening recommendations and that such information could be used to improve online information-seeking and informed decision making about cancer screening.

## Methods

### Data source

We conducted a secondary analysis of 3 years of cross-sectional data from the Health Information National Trends Survey (HINTS) for 2012 through 2014. HINTS is a nationally representative survey, administered annually by the National Cancer Institute since 2003, that collects information on the American public’s use of cancer- and health-related information. Paper questionnaire surveys were mailed to 38,065 households. The HINTS sampling frame is a stratified random sample grouped by US region and by concentration of racial/ethnic minority populations. A detailed description of sampling strategies and methodology can be found on the HINTS website (https://hints.cancer.gov/dataset.aspx). We collected data on 4,537 respondents, 2,067 men and 2,470 women. We included all non-Hispanic whites and non-Hispanic blacks (specifying a 2-level indicator variable accordingly), but excluded other racial/ethnic groups (eg, Hispanics) because of small sample sizes. Black men have a higher risk of prostate cancer than white men, and black women have a lower risk of being diagnosed with breast cancer than white women but a higher risk of dying from the disease (12,13). We included people aged 40 to 75, because our study focused on people at average risk for breast and prostate cancer; people under age 40 are at lower risk for breast and prostate cancer (12,13). People with a previous history of cancer were excluded because cancer survivors are subject to different screening protocols than those at average risk.

### Measures

**Breast and prostate cancer screening.** Women who responded to HINTS were asked whether they had had a mammogram and when they had their most recent one. Because USPSTF recommends mammography every 2 years, women aged 50 to 75 who said they received their last mammogram within the past 2 years were classified as compliers. Women younger than 50 who received a mammogram were classified as non-compliers. (1,14). Men were asked if they ever had a PSA test. Respondents who said yes were classified as noncompliers to the screening guideline. A dichotomous variable was created for each analytic sample to categorize respondents as compliers and noncompliers.

**Online information-seeking.** Online information-seeking was assessed by using measures of how people search for cancer-related information. HINTS asks respondents whether they search for cancer-related information. They are also asked to identify the source they went to first in searching for cancer-related information. We categorized respondents as online information seekers (seekers) if they reported using the Internet as their primary source of information the last time they looked for cancer-related information, or as non-online seekers (nonseekers) if they answered no to using the Internet or reported using any source other than the Internet when they most recently searched for cancer-related information.

**Covariates in the model.** Covariates included in the analyses were age, general health, physician discussion, race, marital status, education, income, occupation, family cancer history, usual source of care, health insurance, number of physician visits in the past year, and health locus of control. Physician discussion refers to whether respondents’ physicians discussed whether respondents should or should not have a PSA test or whether respondents should or should not have a mammogram. Health locus of control refers to respondents’ perception of their ability to control their likelihood of having cancer (15). Respondents were asked to rate their control over their chance of having cancer on a Likert scale from 1 to 4 with 1 being least likely and 4 being most likely. All covariates were self-reported.

### Statistical analysis

We calculated descriptive statistics for variables of interest for the total sample stratified by sex (male and female), which effectively grouped respondents into those at risk for breast cancer or for prostate cancer. Each sample was split again into groups by recommended age criteria. Women respondents were divided into 2 groups: one recommended for cancer screening (50 y to ≤75 y) and one not recommended for screening on the basis of the age criteria of the USPSTF guideline, which does not recommend screening for women younger than 50. Men were in one group because the guideline in effect during our study recommended against routine PSA screening regardless of age. Both samples were constructed by using HINTS data from 2012 through 2014, in light of the USPSTF guideline for prostate cancer screening released in 2012 and the guideline for breast cancer screening released in 2009.

Our statistical analyses had 2 main components. First, we used multivariable logistic regression analysis to examine the relationship between guideline-adherent screening behavior and online information-seeking, controlling for available covariates that might moderate this relationship. Second, we used the Peters–Belson decomposition analysis approach to explore the robustness of our initial findings in relation to the possible omission of unobserved factors that could account for differences in screening behavior between online seekers and nonseekers.

The Peters–Belson method, also known as the Blinder–Oaxaca decomposition, has been used in economics to look at unexplained variation in outcome variables among different groups, such as wage differences between whites and blacks (16). The Peters–Belson method, as applied here, seeks to investigate and quantify the extent to which the difference in screening rates between seekers and nonseekers can be attributed to online information-seeking. The key difference from the logistic regression model (and a contribution to the analysis) is that this method quantifies the effect of unmeasured variables on the differences in screening rates between online information seekers and non-online seekers (17). The difference in screening rates between seekers and nonseekers can be decomposed into the part explained by the covariates (explained variation) and the part not explained by the covariates (unexplained variation), by estimating a model for only the seekers and then measuring how well the model fits for the nonseekers (17). If we let Observed_seek_ and Observed_non-seek_ be proportions of screening rates (observed in the data) for seekers and nonseekers, we can define the difference in screening rates between seekers and nonseekers, expressed as ∆ (17)

∆ = Observed_seek_ – Observed_non-seek_

The analysis first fitted a logistic regression model for seekers for breast and prostate cancer screening. Covariate values for nonseekers were then inserted into the model to estimate the level of difference in screening behavior between seekers and nonseekers (16). Thus, Expected_non-seek_ is defined as the proportion of nonseekers predicted to have engaged in screening had they been online (that is, if their screening behavior had been in accordance with the model estimated for seekers). The difference in observed screening rates between seekers and nonseekers can be rewritten as:

∆ = Observed_seek_ – Observed_non-seek _= (Observed_seek_ – Expected_non-seek_) + (Expected_non-seek_ – Observed_non-seek_)

The difference between the observed and the expected proportion for nonseekers is a measure of the extent to which the model estimated for seekers does not account for the behavior of nonseekers. In the same way, the percentage of variation in the screening behavior of nonseekers that can be explained by the model estimated for seekers can be defined as (17):

Explained % = [(Observed_seek_ – Expected_non-seek_) / ∆ ] * 100

This unexplained portion represents the net influence of factors not available for inclusion in our analyses that could serve to explain differences in screening behavior between seekers and non-seekers. All analyses were performed in Stata Version 14 (StataCorp LLC) and SAS Version 9.4 (SAS Institute Inc) and incorporated sampling weights to account for the complex survey design elements of the data, nonresponse bias, and sampling bias. The study was approved by the Emory University Institutional Review Board.

## Results

In our sample of 4,537 HINTS respondents, 1,297 (29.0%) were seekers, and 3,240 (71.0%) were nonseekers. Among men, 911 (44%) reported being guideline adherent (ie, did not get a PSA test). Among women younger than 50 (ie, those for whom USPSTF does not recommend mammography screening), 216 (33%) reported having mammograms; for women for whom USPSTF recommends mammography (ie, those aged 50 y to ≤75 y), 1,426 (79%) reported guideline adherence. For all 3 groups, seekers had higher screening rates than nonseekers.

Not adjusting for the influence of covariates, we found a strong association between online information-seeking and cancer screening. Male seekers were more likely to be nonadherent (ie, to get a PSA test) than nonseekers. Their likelihood of reporting having a PSA test was strongly related to online information-seeking (*P* = .001). For women aged 50 to 75, the likelihood of having a mammogram, and thus being guideline adherent, was also related to online information-seeking (*P* = .047), as well as for women younger than 50 for whom USPSTF does not recommend mammography (*P* = .03). Female seekers were more likely to get mammograms than nonseekers, regardless of recommendation (Table 1).

**Table 1:** Demographic Characteristics, Respondents (N = 4,537) to Health Information National Trends Survey Regarding Prostate Specific Antigen (PSA) Test and Mammography Screening, 2012–2014

Characteristic	Men, PSA Test^a^			Women, Mammography Not Recommended^a^			Women, Mammography Recommended^a^		
	Not Screened, N = 911	Screened, N = 1,156	*P* Value	Not Screened, N = 216	Screened, N = 440	*P* Value	Not Screened, N = 388	Screened, N = 1,426	*P* Value
**Seeks cancer-related information online**									
Yes	37	63	.001	17	83	.05	27	73	.03
No	46	54		23	77		37	63	
**Age, y (mean, SD)**	49.94 (0.28)	57.86 (0.39)	NA	44.05 (0.30)	44.97 (0.18)	NA	59.36 (0.55)	60.20 (0.22)	NA
**General health**									
Excellent	45	55	.06	30	70	.36	16	84	<.001
Very good	46	54		41	59		17	83	
Good	54	46		31	69		26	74	
Fair	58	42		28	72		31	69	
Poor	63	37		39	61		45	55	
**Physician discussion^b^**									
Yes	12	88	<.001	31	69	.20	24	76	.44
No	85	15		37	63		22	78	
**Race^c^**									
White	51	49	.79	33	67	.20	24	76	.01
Black	50	50		42	58		15	85	
**Marital status**									
Single	59	41	.005	36	64	.72	29	71	.003
Living with a spouse or partner	48	52		34	66		19	81	
**Education**									
<High school graduate	69	31	.001	44	56	.002	29	71	.004
High school graduate	63	37		56	44		21	79	
Some college	47	53		31	69		29	71	
Undergraduate degree or more	41	59		25	75		14	86	
**Annual income, $**									
<14,999	71	29	.001	41	59	.26	31	69	.001
15,000–34,999	52	48		33	67		36	64	
35,000–49,999	54	46		38	62		27	73	
50,000–99,999	48	52		33	67		14	86	
≥100,000	43	57		23	77		6	94	
**Employment**									
Employed	55	45	.002	35	65	.93	21	79	.59
Unemployed	43	57		34	66		23	77	
**Family history of cancer **									
Yes	49	51	.02	30	70	.09	21	79	.17
No	51	49		43	57		28	72	
**Usual source of health care**									
Yes	45	55	.001	30	70	.04	18	82	.001
No	64	36		44	56		37	63	
**Health insurance**									
Yes	48	52	.004	31	69	.10	19	81	.001
No	62	38		44	56		35	65	
**No. physician visits in past year**									
None	67	33	.001	66	34	<.001	60	40	<.001
1	53	47		34	66		22	78	
2	45	55		38	62		13	87	
3	39	61		24	76		16	84	
4	51	49		41	59		18	82	
≥5	44	56		18	82		17	83	
**Health locus of control^d^**									
Strongly agree	64	36	.002	33	67	.91	34	66	.08
Somewhat agree	59	41		39	61		26	74	
Somewhat disagree	51	49		34	66		23	77	
Strongly disagree	44	56		33	67		18	82	

a According to US Preventive Services Task Force Recommendations, 2012­–2014. Values are percentages unless otherwise indicated.

b Physician discussed benefits and risks of screening with the patient.

c Only non-Hispanic whites and blacks were included to capture race as a risk factor.

d Health locus of control refers to one’s perception of ability to control the likelihood of cancer (18).

After adjusting for covariates, we found no significant relationship between online information-seeking and guideline adherence in breast and prostate cancer screening (Table 2). Guideline non-adherence in PSA screening (ie, having a PSA test) was significantly associated with education and physician discussion. Men with higher education or who had discussion about PSA screening with their physicians were less likely to be guideline adherent.

**Table 2:** Analyses of the Likelihood of Guideline Adherence, Respondents (N = 4,537) to Health Information National Trends Survey Regarding Prostate Specific Antigen (PSA) Test and Mammography Screening, 2012–2014

Characteristic	PSA Test, OR (95% CI)	Mammography, OR (95% CI)	
	Men, 40 to ≤75	Women, 40 to <50	Women, 50 to ≤75
**Seeks cancer-related information online**			
Yes	0.61 (0.31–1.21)	0.80 (0.33–1.90)	1.16 (0.63–2.11)
No	1 [Reference]	1 [Reference]	1 [Reference]
**Physician discussion^a^**			
Yes	0.027^b^ (0.016–0.046)	0.74 (0.46–2.23)	0.73 (0.46–1.17)
No	1 [Reference]	1 [Reference]	1 [Reference]
**Age**	0.91^b^ (0.88–0.94)	0.84^b^ (0.71–0.98)	1.06^b^ (1.02–1.10)
**General health**			
Excellent	1.54 (0.31–7.70)	0.87 (0.076–9.86)	6.39^b^ (1.73–23.66)
Very good	1.59 (0.36–7.04)	0.76 (0.066–8.76)	4.54^b^ (1.47–13.96)
Good	1.79 (0.42–7.67)	0.46 (0.043–4.90)	3.01 (0.90–10.08)
Fair	1.41 (0.24–8.27)	0.40 (0.037–4.32)	2.28 (0.76–6.87)
Poor	1 [Reference]	1 [Reference]	1 [Reference]
**Race^c^**			
White	1 [Reference]	1 [Reference]	1 [Reference]
Black	0.70 (0.33–1.46)	1.46 (0.66–3.26)	2.44^b^ (1.32–4.51)
**Marital status**			
Single	0.78 (0.37–1.62)	1.02 (0.38–2.76)	0.80 (0.49–1.32)
Living with a partner	1 [Reference]	1 [Reference]	1 [Reference]
**Education**			
<High school	1 [Reference]	1 [Reference]	1 [Reference]
High school graduate	0.59 (0.24–1.47)	4.38 (0.38–50.97)	0.81 (0.33–2.04)
Some college	0.24^b^ (0.12–0.48)	1.84 (0.24–13.87)	0.53 (0.22–1.29)
≥College	0.27^b^ (0.11–0.66)	1.10 (0.12–9.91)	0.62 (0.19–2.00)
**Annual income, $**			
<14,999	1 [Reference]	1 [Reference]	1 [Reference]
15,000–34,999	0.76 (0.30–1.96)	0.31 (0.086–1.15)	0.77 (0.42–1.42)
35,000– 49,999	0.61 (0.20–1.89)	0.60 (0.14–2.59)	1.05 (0.51–2.16)
50,000– 99,999	0.56 (0.16–1.98)	0.48 (0.07–3.36)	2.69 (0.97–7.46)
≥100,000	0.58 (0.18–1.91)	0.33 (0.033–3.22)	5.48^b^ (1.53–19.67)
**Employment**			
Employed	1.24 (0.70–2.19)	1.10 (0.28–4.37)	1.33 (0.77–2.29)
Unemployed	1 [Reference]	1 [Reference]	1 [Reference]
**Family cancer history**			
Yes	1 [Reference]	1 [Reference]	1 [Reference]
No	1.15 (0.76–1.73)	1.77 (0.72–4.36)	0.66 (0.40–1.07)
**Usual source of care**			
Yes	1.14 (0.60–2.17)	0.72 (0.35–1.47)	1.58 (0.99–2.53)
No	1 [Reference]	1 [Reference]	1 [Reference]
**Health insurance**			
Yes	0.97 (0.26–3.67)	0.55 (0.18–1.74)	1.40 (0.88–2.23)
No	1 [Reference]	1 [Reference]	1 [Reference]
**No. physician visits in past year**			
None	1 [Reference]	1 [Reference]	1 [Reference]
1	0.97 (0.26–3.67)	0.16^b^ (0.045–0.59)	7.35^b^ (3.40–15.89)
2	0.74 (0.30–1.86)	0.27^b^ (0.09–0.79)	10.67^b^ (4.51–25.24)
3	0.54 (0.20–1.47)	0.19^b^ (0.035–0.99)	9.37^b^ (4.31–20.36)
4	1.06 (0.334–3.29)	0.29^b^ (0.083–1.03)	7.78^b^ (2.97–20.40)
≥5	0.67 (0.23–1.98)	0.11^b^ (0.029–0.38)	12.62^b^ (5.09–31.28)
**Health locus of control^d^**			
Strongly agree	1.94 (0.60–6.31)	0.52 (0.10–2.71)	0.89 (0.36–2.21)
Somewhat agree	1.14 (0.65–2.02)	1.06 (0.40–2.79)	0.82 (0.47–1.42)
Somewhat disagree	0.88 (0.50–1.56)	1.00 (0.49–2.03)	0.82 (0.52–1.29)
Strongly disagree	1 [Reference]	1 [Reference]	1 [Reference]

Abbreviations: CI, confidence interval; OR, odds ratio.

a Physician discussed benefits and risks of screening with the patient.

b Denotes significance at *P* = .05.

c Only non-Hispanic white and blacks were included to capture race as a risk factor.

d Health locus of control refers to one’s perception of ability to control the likelihood of cancer (18).

For women overall, having a physician office visit in the past year was positively associated with having a mammogram regardless of whether they were in the age group recommended for screening. Among women recommended for breast cancer screening, age, income, race, and general health were significantly associated with higher odds of guideline adherence. For black women, having an income at or above $100,000 and health reported as excellent, very good, good, or fair were positively associated with receiving a mammogram. For women not recommended for breast cancer screening (40 y to ≤49 y), age was positively associated with receiving a mammogram.

In decomposition analysis for the logistic regression model (Table 3), the total observed difference in screening rates between seekers and nonseekers was significantly different for each of the 3 groups. The largest difference in screening rates between seekers and nonseekers was among men (9.9%) followed by women younger than 50 (8.8%) and women aged 50 to 75 (6.0%). Overall, most of the differences in screening rates between seekers and nonseekers were explained by the estimated coefficients (from the model estimated for seekers only). For men, two-thirds of the difference was explained by the estimated coefficients. For women, a higher percentage of differences, 82.95% for those not recommended for screening and 85% for those recommended, was explained by the model estimated for seekers only. Peters–Belson analyses indicated that most of the differences in screening rates between seekers and nonseekers are accounted for by the estimated models.

**Table 3:** Peters–Belson Decomposition Results, Respondents (N = 4,537) to Health Information National Trends Survey Regarding Prostate Specific Antigen (PSA) Test and Mammography Screening, 2012–2014

Variable	PSA		Mammogram			
	Men, 40 Age ≤75		Women, 40 Age <50		Women, 50 Age ≤75	
	Coefficient (95% CI)	% of Δ	Coefficient (95% CI)	% of Δ	Coefficient (95% CI)	% of Δ
Total Δ between seekers and nonseekers^a^	9.92 (4.68–15.16)	NA	8.82 (1.99–15.64)	NA	6.00 (1.56–10.45)	NA
Explained part^b^	6.94 (2.24–11.63)	66.67	7.30 (3.33–11.26)	82.95	5.15 (2.93–7.36)	85.71
Unexplained part^c^	2.98 (-0.66–6.62)	33.33	1.52 (-4.93–7.97)	17.05	0.86 (-3.5–5.17)	14.29

Abbreviations: Δ, difference in screening rates; CI, confidence interval; NA, not applicable.

a Seeker is a person who searches the Internet for cancer-related information; nonseeker is a person who does not.

b Proportion of differences in screening rates attributable to online information-seeking.

c Unexplained variation in differences in screening rates.

## Discussion

Online information-seeking is not significantly associated with adherence to USPSTF guidelines after adjusting for multiple factors. This finding is in contrast with a past study that found a significant positive association between online information-seeking and screening rates for the recommended groups, and is of interest because an increasing number of people in the general population access the Internet for health-related information (19,20). The logistic regression analyses results we report provide insight into how other individual or environmental factors appear to be associated with screening decisions.

For women recommended for breast cancer screening, the key factor influencing receipt of a mammogram appears to be individual-level factors and barriers. Women recommended for mammography who had better health status, had a higher annual income, and visited their physician in the past year were significantly more likely to be guideline adherent. For non-recommended women, those who visited their physician in the past year were significantly less likely to be guideline adherent. These results are in line with an earlier study that that identified factors and barriers for adherence in breast cancer screening (18).

For men, physician discussion and education level were significantly and positively associated with having PSA screening. Our analyses identified 2 types of men most likely to be screened: those with high education levels who actively seek preventive services and those whose source of information is solely their physicians.

Additional analyses with the Peters–Belson method provide estimates of how well our logistic regression models performed by quantifying the level of unexplained differences in screening rates between seekers and nonseekers. The decomposition results showed that 33.3% of the difference in PSA screening between seekers and nonseekers was not explained by the covariates in the model estimated for seekers only. For women not recommended for mammography and those recommended, 17.05% and 15.00% of the differences in mammogram rates, respectively, were not explained by their corresponding logistic regression models. The smaller unexplained percentages in differences for mammography than for PSA testing indicate that covariates in the model accounted for higher portions of differences in mammography rates between seekers and nonseekers than those in PSA screening rates.

The χ^2^ tests for the unadjusted association, the logistic regression analyses, and the Peters–Belson analyses for the association between online information-seeking and screening and the decomposition results indicate that a large portion of differences in screening rates derive from individual or physician-related factors rather than online information-seeking. The results indicate that online information-seeking itself does not have a clear effect on screening decisions; rather, factors such as physician visits are significantly associated with screening. Past studies have shown that decision aids and physician-initiated screening discussion significantly influence patient decision making, but to a varying degree for different individuals (8–11,14). In our analyses, physician discussion seemed to be a significant factor for PSA screening, but not for receipt of a mammogram. The number of physician visits was a significant factor for having a mammogram, but not for PSA testing.

It is therefore important to have tailored interventions for people at average risk to maximize the benefits of screening. For prostate cancer, physician discussion seems to be the key driver in the PSA decision making process, as indicated in our study and previous studies (21,22). Physicians should also discuss risks of screening to adequately inform their patients. For breast cancer, a physician visit seems to be the key factor in receiving a mammogram, whether guidelines recommend screening or not. For those recommended for screening, it may be important to ensure that these women regularly visit their physicians for preventive services. For women not recommended for screening in USPSTF guidelines, physicians should inform their patients about both the benefits and risks of mammography. Although resources, including physician time, are limited and other treatment priorities for patients compete, our study and past findings indicate physician encounters are key to delivering guideline-adherent care (8,11,14).

Our study has several limitations. Receipt of mammograms and PSA tests are self-reported in HINTS and therefore subject to error. In addition, we could not identify the intensity of online information-seeking or the source of information. There may be differences in the effects of online information-seeking as a function of both the quantity and quality of information identified. Variations in frequency of online searching and in the information’s scientific quality could influence screening decisions in ways that HINTS cannot capture. We also could not capture and control for variations in cancer risk levels for the individuals included in the study, because of the absence of information in HINTS on clinical factors and family history regarding cancer. The respondent’s underlying risk status is therefore an uncontrolled factor in the analyses; however, the decomposition analyses indicate that unmeasured factors play a limited role in explaining the differences in screening rates between seekers and nonseekers.

Finally, it is important to note that a new draft of the USPSTF recommendation for PSA screening was published in early 2017; for men aged 55 to 69, the previous recommendation against PSA screening was changed to a new recommendation that physicians inform patients of potential benefits and harms of screening (22). Because our study analyzed data from 2012 to 2014, the new recommendation does not influence the findings reported here. However, it will be of considerable interest to see whether this change spurs greater online information-seeking among men about prostate cancer and a stronger connection between such information-seeking and having a PSA test, given the emphasis on informed decision making.
